# Opioidergic tuning of social attachment: reciprocal relationship between social deprivation and opioid abuse

**DOI:** 10.3389/fnana.2024.1521016

**Published:** 2025-01-23

**Authors:** Julia A. Galiza Soares, Samantha N. Sutley-Koury, Matthew B. Pomrenze, Jason M. Tucciarone

**Affiliations:** ^1^Department of Psychiatry and Behavioral Sciences, Stanford University, Stanford, CA, United States; ^2^Nancy Pritzker Laboratory, Department of Psychiatry and Behavioral Sciences, Stanford University, Stanford, CA, United States

**Keywords:** opioid, social isolation, early-life adversity, protracted withdrawal, juvenile isolation, maternal separation, rodent behavior

## Abstract

Individuals misusing opioids often report heightened feelings of loneliness and decreased ability to maintain social connections. This disruption in social functioning further promotes addiction, creating a cycle in which increasing isolation drives drug use. Social factors also appear to impact susceptibility and progression of opioid dependence. In particular, increasing evidence suggests that poor early social bond formation and social environments may increase the risk of opioid abuse later in life. The brain opioid theory of social attachment suggests that endogenous opioids are key to forming and sustaining social bonds. Growing literature describes the opioid system as a powerful modulator of social separation distress and attachment formation in rodents and primates. In this framework, disruptions in opioidergic signaling due to opioid abuse may mediate social reward processing and behavior. While changes in endogenous opioid peptides and receptors have been reported in these early-life adversity models, the underlying mechanisms remain poorly understood. This review addresses the apparent bidirectional causal relationship between social deprivation and opioid addiction susceptibility, investigating the role of opioid transmission in attachment bond formation and prosocial behavior. We propose that early social deprivation disrupts the neurobiological substrates associated with opioid transmission, leading to deficits in social attachment and reinforcing addictive behaviors. By examining the literature, we discuss potential overlapping neural pathways between social isolation and opioid addiction, focusing on major reward-aversion substrates known to respond to opioids.

## 1 Introduction

Opioids are powerful drugs widely used for the management of chronic pain, however, their effects extend beyond pain relief to influence emotional and motivational states. Compulsive drug-seeking behavior, tolerance, and withdrawal are hallmark symptoms of opioid use disorder (OUD), a chronic condition characterized by an individual’s inability to control or limit their use of opioid derivatives (DSM-5, 2013). Repeated opioid use can lead to physical and psychological dependence, thus creating a cycle in which the need to avoid withdrawal drives continued drug use (Shurman et al., 2010). OUD can stem from the misuse of prescription pain relievers, such as oxycodone or morphine, or illicitly manufactured opioids, such as fentanyl and heroin. The number of opioid-related deaths has been on the rise for the past two decades, with more than 9.3 million adults in the United States seeking OUD treatment in 2022 and almost 74,000 opioid-related deaths reported in the same year (NIDA, 2022). Opioid withdrawal is characterized by psychological and neurobiological disturbances that often require long-term interventions, such as medication, behavioral therapy, and continuous support systems (Strang et al., 2020). Hyperkatifeia (intensified sensitivity to negative affective states during withdrawal) amplifies social deficits during protracted opioid abstinence, prolonging challenges like anxiety, irritability, and difficulty in forming or maintaining social connections (Koob, 2022). The relevance of OUD extends beyond the personal struggles of those affected as it poses significant public health and societal challenges, including increased rates of overdose, reduced quality of life, and strain on healthcare resources (Florence et al., 2021; Murphy, 2020). Hence, understanding risk factors for OUD is crucial to predicting vulnerability and preventing the development of this chronic brain disease.

The opioid system is a network of three main G-protein coupled receptors (GPCRs): *δ*-opioid receptors (DORs), *κ*-opioid receptors (KORs), and *μ*-opioid receptors (MORs). While opioid use is initially *μ*-driven, chronic opioid use appears to increasingly engage “anti-reward” neural substrates, including signaling through the KOR and its endogenous ligand, dynorphin. DORs are associated with emotional regulation and motivated behaviors but do not seem to be necessary for drug reward processing (Darcq and Kieffer, 2018). MORs are the primary targets of highly addictive substances like fentanyl and morphine, playing a role in both the therapeutic benefits and the maladaptive effects of these drugs (Matthes et al., 1996; Darcq and Kieffer, 2018). KOR agonists lack abuse potential but are known to cause adverse side effects, such as dysphoria, sedation, and aversion (Dalefield et al., 2022). Enhanced KOR signaling during negative affect states has been reported by several studies, characterizing this receptor as a potential driver of pro-addictive behaviors (Massaly et al., 2016; Bruchas et al., 2010; Darcq and Kieffer, 2018; Solecki et al., 2009; D’Addario et al., 2013). Recent reports have described a role for the endogenous KOR-dynorphin system, linking it to negative affect and social deficits during opioid withdrawal (Pomrenze et al., 2022; Kelsey et al., 2015; Marchette et al., 2021; Chen et al., 2023; Lalanne et al., 2017).

Many risk factors for OUD have been identified, including but not limited to pre-existing chronic psychiatric conditions, previous exposure to opioids, drug availability, family history, and sex (Marshall et al., 2023; Martins et al., 2009; Kendler et al., 2023; Hser et al., 2017; Nazarian et al., 2021; Webster, 2017). However, social factors associated with increased risk of opioid abuse and relapse have only started to be explored. With unprecedented and widespread confinement policies, the COVID-19 pandemic offered a unique opportunity to understand the interplay between social isolation and loneliness in humans and their negative impact on mental and physical health (Sepúlveda-Loyola et al., 2020). Loneliness is the subjective feeling of being alone or disconnected despite the presence of others, while social isolation refers to the objective lack of social interactions and connections (Peplau, 1983). Studies examining the effects of social isolation on young adults during the pandemic showed strong associations between loneliness and emotional disturbances, including anxiety, depression, and decreased feelings of connectedness (Horigian et al., 2021). Increased use of several drugs of abuse, such as alcohol and cannabis, was also reported during the period (Layman et al., 2022; Roberts et al., 2021; Martínez-Mota et al., 2020). Importantly, several sources reported spikes in opioid overdoses and naloxone (NLX) administration across the U.S. during the pandemic, with more than 300,000 overdose deaths registered between 2020 and 2023 (Boehnke et al., 2021; Slavova et al., 2020; Volkow, 2020; Roberts et al., 2021; NIDA, 2022). These studies suggest an important and strong link between social isolation, opioid seeking, and relapse, underscoring the importance of understanding how such mechanisms drive OUD.

Recent reports show that, among OUD patients undergoing maintenance treatment, higher self-reported loneliness scores are positively associated with odds of detecting non-prescribed opioids in drug screens, and consequently, of treatment failure (McDonagh et al., 2020). On the other hand, strong social support is associated with decreased chances of opioid misuse and relapse in adolescents and adults (Kumar et al., 2021; Nguyen and Dinh, 2023; Jesmin and Amin, 2020). Another study described social isolation as a predictor for OUD in older adults, further suggesting the need to reframe addiction as a psychosocial issue beyond the individual level and to adopt social network interventions that improve treatment outcomes (Yang et al., 2022). Interestingly, it appears that not only can social factors influence opioid addiction, but that opioid use promotes increased feelings of social isolation and weakened social functioning. Opiate users in maintenance therapy showed reduced capacity to identify facial expressions and assess social situations (McDonald et al., 2013; Kornreich et al., 2003; Martin et al., 2006). Studying this apparent bidirectional relationship is essential, as social isolation may both exacerbate addiction vulnerability and be a consequence of addictive behaviors. This narrative review will examine how social factors modulate brain regions, circuits, and systems implicated in OUD and the role of opioid signaling in social bonding and attachment formation. Moreover, we will explore the interaction between these mechanisms to pinpoint the possible shared neural substrates between social distress and opioid withdrawal. Lastly, we will highlight critical gaps in the field and suggest how future studies may further explore the link between social isolation and opioid addiction.

## 2 Opioid substrates underlying attachment formation and sociability

Attachment theory, developed by psychiatrist John Bowlby and psychologist Mary Ainsworth, explores how early emotional bonds between infants and their caregivers shape an individual’s ability to form and maintain relationships throughout life (Ainsworth and Bowlby, 1991). Their work set the foundation for understanding the need for children to bond with primary caregivers for healthy social, cognitive, and emotional functioning in adulthood (Wade et al., 2022). Parent-infant attachment provides care and protection and increases offspring survival and lack of parental care and nurturance can lead to several challenges in adulthood, including psychiatric and medical disorders (Lippard and Nemeroff, 2023). Although the attachment system is initially organized around primary caregiving figures, it later expands to include intimate partners and friends (Bowlby, 1977). This bond is theorized to result from evolutionarily conserved opioid circuits that encode pain and reward, which may in turn promote opioid release during certain types of social interactions (Panksepp et al., 1994). The brain opioid theory of social attachment posits that endogenous opioids—natural brain neuropeptides involved in pleasure and reward—play a crucial role in forming and maintaining social bonds by reinforcing positive social interactions. In this framework, social isolation is theorized to reduce endogenous opioid release and promote distress (Herman and Panksepp, 1978). On this basis, disruptions in the opioid system, such as those caused by opioid addiction, are thought to impair the ability to experience social rewards, leading to difficulties in maintaining close relationships and increased social isolation. In this way, the role of the endogenous opioid system in bond formation and attachment may explain the apparent bidirectional causal relationship between social isolation and opioid addiction.

Growing evidence suggests a shared neural basis between physical pain and “social pain”—emotional suffering or distress that arises from social interactions or their lack thereof (Eisenberger, 2012). Early pharmacological studies show that low morphine doses can reduce distress vocalizations in socially isolated pups of several species (Panksepp et al., 1978; Herman and Panksepp, 1978; Kalin and Shelton, 1989; Kalin et al., 1988), which can, in turn, be increased by NLX, a MOR antagonist (Herman and Panksepp, 1978; Carden et al., 1996; Martel et al., 1995; Panksepp et al., 1980; Kalin et al., 1988). Repeated administration of another opioid antagonist, naltrexone (NTX), during infancy decreases interest in peers and preference for socially rewarding environments in juvenile rodents (Cinque et al., 2012; Smith et al., 2015). Intra-nucleus accumbens (NAc) *β*-endorphin and morphine infusions enhance play behavior that can be blocked by NLX administration (Trezza et al., 2011). Thus, opioid signaling appears to play a crucial role in modulating social behaviors, influencing factors such as attachment and social reward, which can be atypical in individuals with autism. Mice lacking the MOR gene (*Oprm1*^−/−^) have been established as a monogenic mouse model of autism (Becker et al., 2021). MOR-KO mice show decreased social preference for their mothers and no distress vocalization following maternal separation, further supporting the role of *μ*-signaling in attachment formation (Cinque et al., 2012; Moles et al., 2004). The number of opioid receptor gene copies also appears to influence affiliative behavior, with *Oprm1*^+/−^ mice having reduced social preference and interest in potential sexual partners (Toddes et al., 2021). Not only do *Oprm1*^−/−^ show social deficits, but also motor stereotypies, altered nest-building behavior, and anxiety-like behaviors (Becker et al., 2021). These results suggest that endogenous opioids may be released during prosocial behavior, binding to MORs to influence social reward and positively modulate social behavior. The social-opioid model proposes a parallel between social and drug dependence, with emotional distress after attachment loss analogous to a withdrawal state that could be alleviated through opioid release (Panksepp, 1998). Further studies are needed to elucidate the influence of opioid signaling on the neural circuitry of attachment formation and affiliative behavior.

Pharmacological manipulation of opioid signaling in clinical studies further highlights the role of this system in social experiences. In a recent study, morphine (*vs.* NTX) administration increased attractiveness ratings of opposite-sex faces, as well as the motivation to circumvent conventionally unattractive faces (Chelnokova et al., 2014). Moreover, morphine has been shown to increase the total time spent exploring faces, suggesting that *μ*-signaling influences social interest (Chelnokova et al., 2016). In this way, opioid activation and inhibition appear to enhance hedonic salience (“liking”) and reduce the motivational salience (“wanting”) of social stimuli, respectively. Another study reported NTX-associated decreases in ratings of social connection in response to positive messages from loved ones, with antagonist use also correlated with reduced feelings of connectedness in real-life settings (Inagaki et al., 2016; Charles et al., 2020; Inagaki et al., 2019). In addition, positron emission tomography PET data suggests that social laughter in humans increases endogenous opioid release in several brain regions, including the anterior insula, the caudate nucleus, and the thalamus (Manninen et al., 2017; Sun et al., 2022). In another study, Manninen et al. reported increased opioid release in individuals exposed to social laughter, as well as a decreased pain threshold (Manninen et al., 2017). Variance in basal *μ*-signaling has also been associated with differences in affiliative behavior and attachment in humans (Turtonen et al., 2021; Nummenmaa et al., 2015). On the other hand, depressed states have been linked to the deactivation of *μ*-transmission in key emotional regulation substrates, such as the amygdala and the rostral anterior cingulate cortex (ACC) (Zubieta et al., 2003; Nummenmaa et al., 2020). Greater MOR activation in the amygdala, subgenual ACC, and periaqueductal gray (PAG) has been correlated with greater resilience to social rejection, suggesting that opioid signaling counteracts social distress-induced neural activation in these substrates (Hsu et al., 2013). Hence, it appears that endogenous opioids are released upon social interaction and serve to alleviate social distress. Meanwhile, opioid antagonism may work to enhance social distress behaviors while altering the hedonic value of social interactions.

Clinical studies have also explored the effects of *Oprm1* gene variations on social behaviors. Although more than a hundred single nucleotide polymorphisms (SNPs) of this gene have been reported, the biological function of each variant remains unclear (Mura et al., 2013). A119G *Oprm1* polymorphism has been correlated with enhanced sensitivity to social rejection, with G allele carriers showing increased neural activity in the dorsal ACC and anterior insula, both of which are to be involved in pain processing (Way et al., 2009). In a recent study, G-allele carriers reported a statistically higher likelihood of engaging in affectionate interpersonal relationships and experiencing pleasure derived from social interactions, as compared to A-carriers, further highlighting a potential role of opioid signaling in facilitating affiliative behavior (Troisi et al., 2011). The link between A118G polymorphism and enhanced social pain sensitivity is consistent with human data linking another *Oprm1* variation, A118G, with increased infant separation distress (Tchalova et al., 2024). In this study, the presence of A11G8 polymorphism was linked with developing an ambivalent (*vs*. secure) attachment style in response to maternal insensitivity. Ambivalent attachment is a type of insecure attachment characterized by anxiety and uncertainty about the caregiver’s availability and responsiveness, leading to (1) attachment system hyperactivation with an increase in distress behaviors such as crying and clinginess or (2) attachment system deactivation and social withdrawal. Children carrying the mutant G-allele showed heightened separation distress in response to maternal insensitivity compared to those without the genetic variant. Another study reported that G-carrying mothers tended to showcase higher maternal insensitivity than A/A mothers, as well as increased hostility (e.g., aggressive or antagonistic behaviors or attitudes) toward others (Cimino et al., 2020). The G-carrying children were reported to exhibit increased social withdrawal (Cimino et al., 2020). While several associations have been reported correlating with A119G polymorphisms, the behavioral phenotypes can be diverse and inconsistent study to study so caution must be applied attributing causality to any single mutation. At the same time, these studies reinforce the role of opioid transmission in attachment bond formation and prosocial behavior, with fluctuations of endogenous opioids possibly driving the experience of social reward or distress in response to relational cues.

## 3 Consequences of early social isolation in addiction vulnerability

Early social bond formation and social environment are of enormous importance to development, having long-lasting impacts on brain function and behavior. Several clinical studies support a strong correlation between childhood abuse and opioid use later in life (Carlyle et al., 2021; Lovallo et al., 2018; Conroy et al., 2009; Heffernan et al., 2000; Nelson et al., 2006; Austin et al., 2018). Particularly, childhood neglect has been linked with an increased probability of opioid abuse in adulthood (Austin and Shanahan, 2018). It is estimated that roughly 40% of OUD patients have a history of physical or emotional neglect (Santo et al., 2021; Austin and Shanahan, 2018). The mechanisms through which early-life adversity (ELA) increases vulnerability to OUD remain under investigation, however, existing literature provides multiple avenues to be explored. Relevant to this review are the potential effects of ELA on reward circuitry maturation and function. ELA is thought to modify the function of several brain regions relevant to affective behaviors, cognition, and reward processing, including the amygdala (Tottenham et al., 2011; Hein and Monk, 2017), the prefrontal cortex (PFC) (Hodel et al., 2015; Hanson et al., 2010; De Brito et al., 2013), and the ventral striatum (Goff et al., 2013; Hanson et al., 2015). Moreover, it is plausible to hypothesize that childhood adversity may increase vulnerability to opioid abuse through disruption of opioidergic transmission. As previously discussed, rodent and human data suggest that the opioid system influences attachment formation in early-life dynamics. In this context, disruptions in social development, such as impaired bonding or social isolation, could influence the expression and function of the opioid system and increase the risk of opioid abuse. For this review, we will explore how ELA influences the function of brain reward centers, the opioid system, and the consumption of opioids, with a particular focus on three rodent models of ELA: juvenile social isolation (JSI), maternal separation (MS), and limited bedding and nesting (LBN).

### 3.1 Juvenile social isolation

JSI involves separating young mice from their conspecifics during a critical developmental period, typically post-weaning, to study the effects of social deprivation on behavior, neurodevelopment, and stress-related responses. JSI is known to generate several behavioral deficits in rodents, including anhedonia, distress- and anxiety-like behaviors, sleep disturbances, pain, and social deficits, many of which are also present during protracted opioid withdrawal states (Table 1). This paradigm models deficits in social life during a critical developmental period for establishing learning and memory processes in rodents (Einon and Morgan, 1977). The mesolimbic dopamine (DA) pathway is particularly relevant to understanding maladaptive learning that promotes drug-seeking behaviors (Kauer and Malenka, 2007). Opioid receptors have long been thought to modulate both the hedonic experience and the aversive aspects of drug use beyond those belonging to the opioid class (Herz, 1997). In fact, growing literature provides support to the importance of opioid-DA interactions within the brain reward system that are thought to amplify the motivational drive to continue drug use, establishing a cycle of dependence and vulnerability to relapse (Fields and Margolis, 2015; Volkow, 2010; Trigo et al., 2010). MOR activation enhances DA release in the NAc, reinforcing drug-seeking behavior, while KOR activation can inhibit DA release, contributing to dysphoria and stress-related relapse (Bruijnzeel, 2009; Contet et al., 2004). An in-depth discussion of such mechanisms involving DA-opioid interactions of various substances lies beyond the scope of this review and have been incompletely investigated in animal research (see Le Merrer et al., 2009; Volkow, 2010; Berrendero et al., 2010; Gerrits et al., 2003; Volkow et al., 2019, for comprehensive reviews on this topic).

**Table 1:** Summary table of negative affective behaviors that have been reported in rodents following JSI and protracted spontaneous withdrawal from protracted opioid withdrawal.

	Juvenile social isolation	Protracted withdrawal
Anhedonia	Decreased SP (Du Preez et al., 2020)	Reduced FPP (Harris and Aston-Jones, 2003)
Anxiety-like	Decreased OF center time (Ieraci et al., 2016; Du Preez et al., 2020; Kumari et al., 2016; Kaushal et al., 2012; Haj-Mirzaian et al., 2019) Increased NSFT latency (Du Preez et al., 2020) Decreased EPM open time (Kumari et al., 2016; Kaushal et al., 2012) Increased NIH latency (Zhang et al., 2012)	Decreased OF center time in females (Bravo et al., 2020) Decreased EPM open time (Fox et al., 2023)
Distress-like	Increased TST and/or FST immobility (Du Preez et al., 2020; Haj-Mirzaian et al., 2019; Ieraci et al., 2016)	Increased TST and/or FST immobility time (Listos et al., 2022; Goeldner et al., 2011; Lutz et al., 2014)
Social behavior	Increased aggression in males (Wang et al., 2022; Tan et al., 2021) and social withdrawal in females (Wang et al., 2022; Kuniishi et al., 2022)	Decreased social interaction time (Fox et al., 2023; Bravo et al., 2020; Pomrenze et al., 2022; Becker et al., 2021; Goeldner et al., 2011)
Pain	Increased TF pain threshold (Tuboly et al., 2009)	Mechanical hypersensitivity (McDevitt et al., 2021)

For behavioral consequences of MS (see Supplementary Table S1). SP, sucrose preference; FPP, food place preference; OF, open-field; NSFT, novelty-suppressed feeding test; EPM, elevated-plus maze; NIH, novelty-induced hypophagia; TST, tail suspension test; FST, forced-swim test; TF, tail-flick.

JSI has been shown to impact the development and function of the mesolimbic DA pathway, which may have broad impacts on opioid reward. For example, JSI has been demonstrated to produce an irreversible enhancement of glutamatergic N-methyl-D-aspartate (NMDA) receptor-dependent long-term potentiation (LTP) in the ventral tegmental area (VTA), similar to changes induced by repeated amphetamine exposure (Whitaker et al., 2013). Moreover, alcohol and amphetamine-associated contextual memory were acquired faster and were more resistant to extinction. Another study has reported excitability changes in NAc medium spiny neurons (MSNs), with socially isolated mice exhibiting neuronal hyperactivity as shown by increased excitability, membrane resistance, and presynaptic release probability (Zhang et al., 2019). Other reports indicate that NAc MSNs exhibit morphological differences after JSI, showing reductions in total dendritic length, which can lead to differences in MSN physiological properties (Wang et al., 2012). Animals reared in social isolation also exhibit enhanced DA response in the NAc to both aversive and rewarding stimuli, with subthreshold intensity stimulation eliciting more DA release in JSI rats than controls (Karkhanis et al., 2019). In male rats, JSI blunted reward anticipatory DA release in the medial PFC (mPFC) but increased DA release in the NAc shell during reward consumption (Lallai et al., 2024). The same study further demonstrated that JSI mice showed a robust potentiation of reward-induced extracellular DA release after 2 weeks of reward consumption compared to the first day of reward consumption, suggesting that changes in motivated behavior and reward-seeking may be influenced by DA tone. Overall, these studies indicate that JSI could potentiate DA tone and sensitivity to rewards, which could increase the susceptibility to drug reinforcers and the development of drug-seeking behavior.

Growing literature provides evidence for the lasting effects of social isolation rearing in addiction vulnerability. JSI male rats exhibited enhanced anxiety-like behaviors and stronger morphine conditioned place preference (CPP) (Yazdanfar et al., 2021). These effects may be due to increased motivation for seeking opioid rewards. In a recent study, JSI mice trained to self-administer heroin in a progressive ratio (PR) schedule showed increased motivation and enhanced cue-induced reinstatement (Singh et al., 2022). JSI rats also showed increased morphine consumption in a one-bottle forced-consumption test, an effect that was abolished with as little as 1 h of daily social interaction (Marks-Kaufman and Lewis, 1984). Additional evidence suggests that socially isolated rats learn to self-administer heroin more rapidly than group-housed controls (Bozarth et al., 1989). Social isolation also affects morphine-induced behavioral sensitization, with socially isolated rats showing increased locomotor responses to morphine, an effect blocked by inhibition of corticosterone secretion (Deroche et al., 1994). In comparison, rats raised in enriched environments with exposure to novel objects and conspecifics exhibit reduced remifentanil intake compared to socially isolated rats and controls (Hofford et al., 2017). Altogether, the data describing the effects of early social isolation on the opioid system and opioid reward are limited, and further investigation is required to understand why individuals exposed to early-life social isolation may be at an increased risk for substance abuse, particularly OUD.

Given the well-established roles of the opioid system in several of the circuits underlying the maladaptive behaviors exhibited by JSI rodents, as well as the previously discussed role of opioid signaling in early attachment formation, recent studies have attempted to address long-term changes in endogenous opioids and opioid receptor expression following adolescent social isolation. A 2016 study provided evidence to suggest increased dynorphin levels in the NAc core and shell of JSI rats reduced baseline DA levels (Karkhanis et al., 2016). As discussed previously here, KORs have an important role in modulating mesolimbic DA signaling, with select negative affective states enhancing KOR function and affecting both DA and serotonin (5-HT) release in the NAc. JSI stress has been reported to decrease MOR and KOR gene expression, particularly in the hippocampus and amygdala (Haj-Mirzaian et al., 2019). Additionally, the expression of opioid peptides appears to be influenced by early-life social isolation, with the effects varying based on the length of the isolation period. Whereas prolonged JSI reduced the expression of met-enkephalin in the PFC, substantia nigra, amygdala, hypothalamus, and PAG, short single-housing episodes increased the expression of nociceptin/orphanin FQ in the mPFC, NAc, and amygdala (Granholm et al., 2015). Such reductions in met-enkephalin levels in key areas of reward and stress circuits further suggest disturbances in opioid circuitry development and maturation.

### 3.2 Maternal separation and limited bedding and nesting paradigms

MS involves temporarily splitting rodent pups from their mother to model early-life stress, while the LBN paradigm restricts nesting materials to induce fragmented maternal care. Both are used to model the long-term effects of fragmented and unpredictable maternal care in rodents. MS has been shown to disrupt the development and function of neural circuits involved in reward processing, particularly those associated with DAergic signaling, potentially increasing susceptibility to addiction-related behaviors (Gracia-Rubio et al., 2016; Der-Avakian and Markou, 2010; Sasagawa et al., 2017; Parks et al., 2024). Literature reports changes in tyrosine hydroxylase (TH) and D1 receptor (D1R) mRNA levels in the VTA of female adult mice that underwent MS during postnatal days 1–14 (P1-14), as well as hypermethylation of the D1R promoter region in the NAc (Sasagawa et al., 2017). A recent study further provides evidence for long-lasting transcriptional and transcriptome changes following ELA, with MS exhibiting VTA chromatin modifications associated with stress hypersensitivity (Geiger et al., 2024). Moreover, LBN has been reported to alter connectivity in the dorsal raphe nucleus (DRN)-VTA-NAc pathway and cause negative affect and social deficits in adulthood (Wendel et al., 2021). Another study showed that MS reduced D2 receptor (D2R) protein levels in the frontal cortex and NAc but increased protein levels of TH, further suggesting that DA synthesis and signaling may be altered by MS (Romano-López et al., 2016). ELA also may exert its effects through changes in neuronal morphology. MS has been shown to reduce dendritic spine density in PFC and NAc neurons and the length of dendrites in the PFC, NAc, and hippocampus of adult rats (Monroy et al., 2010). MS has been reported to disrupt the balance of D1/D2 receptor-expressing cells in the prelimbic PFC (plPFC), increasing the density of D1R expression during adolescence while decreasing the density of D2R expression during adolescence and into adulthood (Brenhouse et al., 2013). Moreover, inhibition of plFC D1Rs with antagonist blocks both heroin-primed and cue-induced reinstatement of heroin-seeking in rats (Capriles et al., 2003). Distinct mPFC→NAc projections have been reported to play important roles during opioid-seeking, being hyperactivated during drug relapse (Clarke et al., 2024). In the same study, optogenetic inhibition of corticostriatal projections was shown to decrease heroin-seeking. Given the well-known importance of PFC signaling in opioid-seeking behaviors (Van den Oever et al., 2008; Bossert et al., 2011), more specifically in learning associations between opioid reward and associated context, such morphological and receptor level changes are likely to affect opioid-related learning and behaviors.

Several studies provide evidence that these paradigms can alter the rewarding properties of opioid agonists while enhancing the aversiveness of antagonists. Daily MS episodes during postnatal days 1–14 (P1-14) have been shown to lower the drug dosage threshold, increase the time required to extinguish morphine-induced CPP in rats, and enhance time spent in the morphine-paired chamber (Vazquez et al., 2005; Michaels and Holtzman, 2008). Additionally, MS offspring demonstrated enhanced sensitivity to KOR agonism, as illustrated by increased aversion in the conditioned place aversion (CPA) assay (Michaels and Holtzman, 2008). Repeated deprivation also increased drug-seeking behavior, suggesting that MS episodes enhanced both morphine reward memory and consumption. Another study suggests that these findings are sex-dependent, with males exhibiting stronger morphine CPP and enhanced sensitivity to NTX in a NLX-induced suppression of drinking test (Michaels and Holtzman, 2007, 2008). LBN has been shown to promote heroin-seeking behavior in rats, as demonstrated by impaired extinction and enhanced reinstatement during PR self-administration (Leyrer-Jackson et al., 2021). Additional evidence suggests that ELA might affect males and females differently, as LBN appears to increase opioid-seeking in females but decreases in males (Levis et al., 2022b; Levis et al., 2021). Hyperlocomotion in response to morphine has also been observed in MS rats (Kalinichev et al., 2002).

ELA effects on addiction vulnerability may also be explained by persistent changes in the expression of opioid-related signaling genes due to stressful early-life environmental conditions. In mice, P15-21 MS for 6-h periods reduced MOR/KOR/DOR mRNA expression in the PAG, an area that is central to mediating descending pain control, but increased KOR levels in the amygdala (Nakamoto et al., 2020). Increased MOR and KOR mRNA levels in areas critical to opioid rewards, including the hippocampus, PFC, and NAc have been reported in adult male rats following MS (Askari et al., 2022). A recent study reported LBN-induced reductions in DOR mRNA levels in the NAc (Levis et al., 2024). Pharmacological manipulation of NAc DORs characterized a possible role for this receptor subtype in ELA-induced opioid vulnerability, as DOR blockade was shown to reduce motivation to consume opioids in LBN females. Another study reports increased opioid receptor gene expression in the dorsal striatum for all three subtypes in rats exposed to the MS paradigm (Martin et al., 2006). Furthermore, lower striatal expression of KORs and DORs in rats exposed to ELA was correlated with enhanced ethanol drinking. Bridging and translating these results to the role of ELA and OUD in humans, an important recent human postmortem study reported strong correlation between childhood abuse and long-lasting epigenetic changes in KOR expression, as shown by downregulation of the receptor and reduction of DNA hydroxymethylation (Lutz et al., 2018). However, there is conflicting evidence that opioid peptide levels themselves may be affected by MS. While one group reported that there were no differences in the levels of dynorphin or met-enkephalin in MS rats compared to controls (Marmendal et al., 2004), another reported significant differences in both peptides spanning several brain areas in a manner that was dependent on age, duration of MS, and whether pups were isolated from their littermates during MS sessions or kept together (Gustafsson et al., 2008). Given the many variables involved in the MS protocol, including the duration of each MS session, group *vs* complete isolation, as well as the total number of separation episodes, studies have reported inconsistent behavioral outcomes (see Supplementary Table S1). While early studies report the emergence of abnormal behavioral phenotypes following MS, recent literature suggests that those effects are not as robust as initially thought. This suggests that the MS model might yield more inconsistent results in modeling ELA in mice in comparison to other species in which the model is well-established, such as rats and primates (Holmes et al., 2005; Pryce et al., 2002).

The literature discussed provides evidence that ELA disrupts reward circuit development and increases vulnerability to OUD, however this effect may not be specific to opioids. Indeed, increased addiction vulnerability caused by early social isolation has also been observed in response to other drugs of abuse such as alcohol (Panksepp et al., 2017), cocaine (Fosnocht et al., 2019), and methamphetamine (Webb et al., 2022). While the exact mechanism behind this phenomenon remains underexplored, changes in endogenous opioid transmission following ELA may offer a plausible explanation. Imaging studies have reported changes in opioid binding in brain reward regions in cocaine-dependent individuals, suggesting an important role for opioid signaling in drug reward (Zubieta et al., 1996; Ghitza et al., 2010). In rodents, NTX has been shown to decrease cocaine-seeking and -induced reinstatement (Burattini et al., 2008). Buprenorphine, a partial MOR agonist and KOR antagonist, has been shown to reduce cocaine consumption in non-human (Mello et al., 1989) and human primates (Montoya et al., 2004; Schottenfeld et al., 1993; Kosten et al., 1989). Several studies have also explored the role of the opioid system in alcohol consumption. Knock-out of the three opioid receptor subtypes has been shown to alter ethanol self-administration (Roberts et al., 2001; Kovacs et al., 2005; Roberts et al., 2000). Moreover, alcohol consumption can be altered by opioid antagonists such as NLX and NTX, with the latter being widely used in the treatment of alcohol use disorder (Volpicelli et al., 1992). Given the proposed role of endogenous opioid signaling in reward processing, changes in the opioid system following early social isolation could broadly impact drug-related behaviors.

## 4 Shared neural underpinnings of social deprivation and opioid withdrawal

As discussed, impaired social bond formation and poor social environments in early life can have long-lasting impacts on brain function and behavior. In the past two decades, studies have characterized the long-term consequences of early social deprivation, which include anxiety- and depressive-like behaviors, as well as sleep disturbances and social deficits (Table 1). Growing literature describes the brain opioid system as a powerful modulator of social separation distress and attachment formation in rodents and primates (Lutz et al., 2020). Preliminary studies have linked changes in endogenous opioid peptides and receptors in ELA models with increased vulnerability to opioid addiction (Levis et al., 2022a). While social deprivation appears to increase vulnerability to opioid abuse, opioid use is also thought to disrupt social functioning, leading to withdrawal from social interactions and difficulties in maintaining interpersonal relationships (McDonald et al., 2013; Kornreich et al., 2003; Martin et al., 2006; McDonagh et al., 2020). Moreover, protracted opioid withdrawal is similarly marked by negative emotional states, including stress, anxiety, and dysphoria (Ozdemir et al., 2023). Remarkably, mechanical hyperalgesia is known to occur both following opioid abstinence (Yi and Pryzbylkowski, 2015) and early social deprivation (Weaver et al., 2007; Vilela et al., 2017; Amini-Khoei et al., 2017; Paniagua et al., 2020). Given the well-established role of opioid signaling in pain processing, ELA-induced pain sensitivity offers further evidence for disruptions in opioid circuit development in response to stressful environmental conditions and improper attachment formation in early life. Finally, social deficits have been reported during opioid withdrawal (Fox et al., 2023; Bravo et al., 2020; Pomrenze et al., 2022; Becker et al., 2021; Goeldner et al., 2011), as well as after early social deprivation (Wang et al., 2022; Kuniishi et al., 2022; Tan et al., 2021).

**Figure 1:**
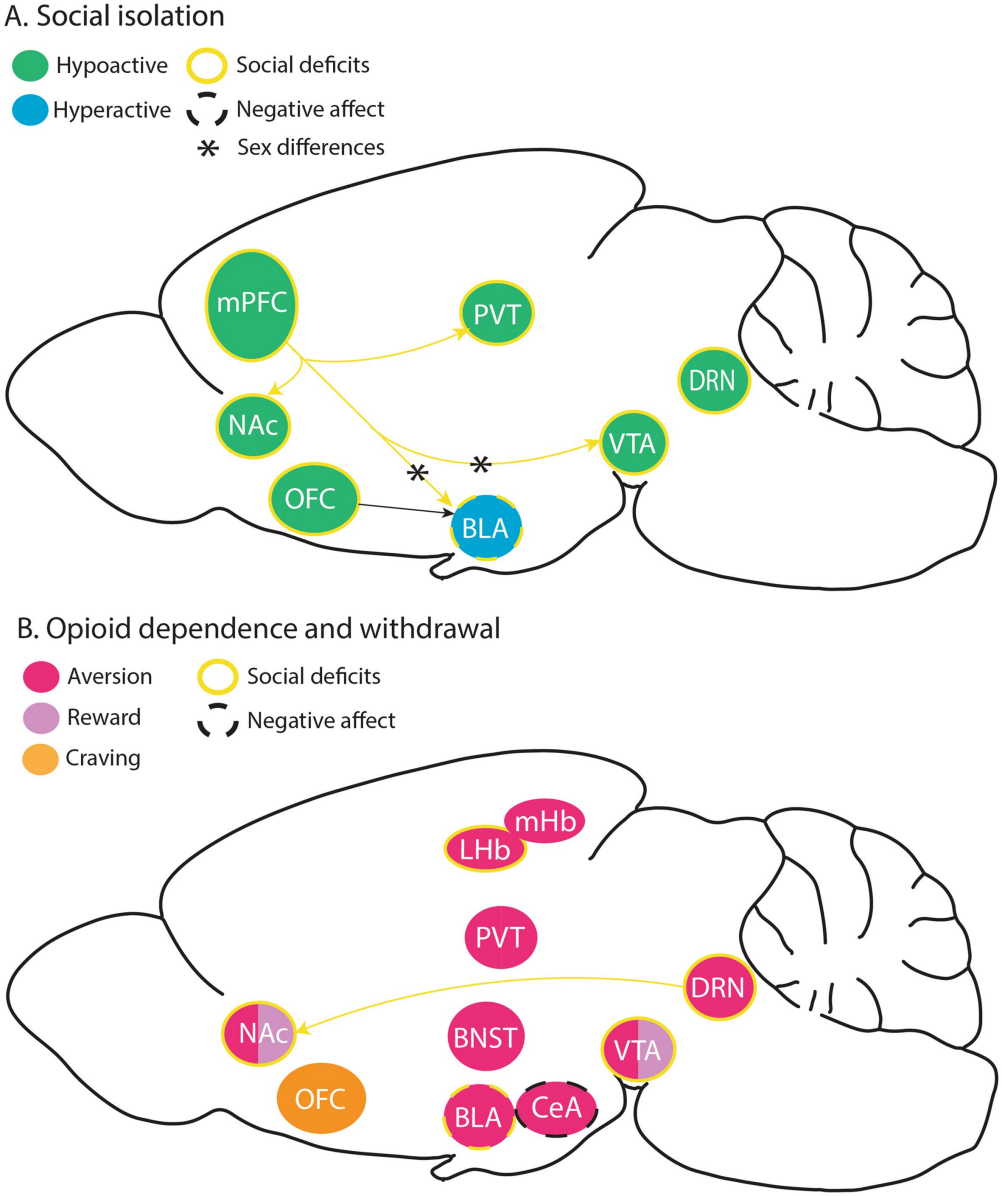
Brain substrates modulating social isolation- and opioid withdrawal-induced negative affect, as well as circuits underlying social deficits in these rodent models. **(A)** Hypo and hyperactive brain substrates thought to facilitate negative effect and social deficits following social isolation, with some known sex differences. **(B)** Brain substrates thought to play a role in aversive and rewarding effects of opioids, opioid withdrawal-induced social deficits, negative affect, and craving. BLA, basolateral amygdala; BNST, bed nucleus of the stria terminalis; CeA, central amygdala; DRN, dorsal raphe nucleus; LHB, lateral habenula, MHb, medial habenula; mPFC, medial prefrontal cortex; NAc, nucleus accumbens; OFC, orbitofrontal cortex; PVT, paraventricular thalamus; VTA, ventral tegmental area.

The net effect of opioid receptor signaling on neural circuit activity depends on the cell types that express the receptor and its subcellular localization. Opioid receptors are particularly enriched in areas involving stress, emotion, pain, and reward processing, including but not limited to the thalamus, habenula (Hb), amygdala, cortex, striatum, and monoamine nuclei (Arvidsson et al., 1995; Mansour et al., 1988). MOR expression is highest in the Hb and thalamic nuclei, whereas KOR is prominent in the hypothalamus and hippocampus (Lutz et al., 2014). This section will discuss the current literature on the brain substrates underlying long-term effects of early social deprivation and opioid withdrawal, focusing on potential shared neural underpinnings between social impairment and opioid use (Figure 1).

### 4.1 Monoamine nuclei

The role of mesolimbic DA in mediating the rewarding effects of opiates is well-established (Di Chiara and Imperato, 1988), however, its influence on withdrawal states remains underexplored (Table 2). Studies have reported long-lasting alterations of DAergic VTA (VTA^DA^) neuron signaling during opioid abstinence (Diana et al., 1995, 1999; Georges et al., 2006; Kaufling and Aston-Jones, 2015). Administration of acute morphine injections fails to change the activity of this population during protracted opioid withdrawal (Georges et al., 2006). This tolerance following opioid cessation appears to be partially mediated by reduced glutamatergic inputs in the VTA (Kaufling and Aston-Jones, 2015). Both optogenetic activation of GABAergic VTA neurons and inhibition of VTA^DA^ neurons can produce CPA, further suggesting that VTA activity could modulate aversive behavioral states during opioid withdrawal (Tan et al., 2012).

**Table 2:** Summary table of changes in brain activity and function following opioid abstinence.

Region/system	Role in opioid dependence and withdrawal
BLA	Negative affective signs (Deji et al., 2022; Zhu et al., 2023), and drug withdrawal memory (Guo et al., 2024)
BNST	Drug-seeking behavior (Kudo et al., 2014; Beckerman and Glass, 2012; Delfs et al., 2000; Gyawali et al., 2020)
CeA	Somatic symptoms of precipitated withdrawal (Chaudun et al., 2024), and negative affective signs (Jiang et al., 2021)
DRN	Hyperalgesia (Alvarez-Bagnarol et al., 2023), propensity to relapse (Welsch et al., 2023), and social deficits (Lutz et al., 2014; Pomrenze et al., 2022)
LHb	Social deficits (Valentinova et al., 2019)
MHb	Somatic withdrawal signs (Boulos et al., 2020)
NAc	Reward processing (Cui et al., 2014; Castro et al., 2021), withdrawal behavior and aversion (Zhu et al., 2023)
OFC	Incubation of craving (Fanous et al., 2012; Sun et al., 2006)
PVT	Somatic withdrawal signs and drug withdrawal memory (Zhu et al., 2016), suppression of reward-seeking (Vollmer et al., 2021), and opioid-induced sleep disturbances (Eacret et al., 2023)
VTA	Rewarding effects and reinforcement of drug-seeking behavior (Zhang et al., 2009; Di Chiara and Imperato, 1988; Pierce and Kumaresan, 2006), opiate tolerance (Kaufling and Aston-Jones, 2015), and social deficits during protracted withdrawal (Jo et al., 2024)

BLA, basolateral amygdala; BNST, bed nucleus of the stria terminalis; CeA, central amygdala; DRN, dorsal raphe nucleus; LHB, lateral habenula, MHb, medial habenula; NAc, nucleus accumbens; OFC, orbitofrontal cortex; PVT, paraventricular thalamus; VTA, ventral tegmental area.

Studies have started to describe the role of distinct MOR-expressing neuronal populations in mediating opioid-seeking behavior and reward. The VTA and the DRN contain MOR-expressing neuron populations that seem to facilitate both reward and aversion processing associated with chronic opioid use (Jo et al., 2024; Welsch et al., 2023; Lutz et al., 2014; Fields and Margolis, 2015). MOR knockdown in the VTA disrupts heroin CPP, suggesting that VTA *μ*-activity is necessary for the rewarding effects of opioids (Zhang et al., 2009). Morphine abstinence has been reported to impair MOR-expressing DRN neurons via the downregulation of genes relevant to synaptic signaling, leading to abnormal responses to opioids (Welsch et al., 2023). These neurons have also been implicated in opioid-induced hyperalgesia (Alvarez-Bagnarol et al., 2023). Moreover, several studies support that chronic opioid use increases 5-HT activity, suggesting interactions between serotonergic and opioidergic signaling (Wei et al., 2018; Tao and Auerbach, 2002). Specifically, a recent study has shown that KORs block 5-HT release in the NAc during morphine protracted withdrawal, contributing to the development of social deficits (Pomrenze et al., 2022).

Several studies have reported that positive social interactions engage some of the same brain substrates involved in drug reward processing. Notably, the activity of VTA^DA^ neurons projecting to the NAc predicts social interaction, with increased neural firing in the NAc causally linked to higher social exploration in freely behaving mice (Gunaydin et al., 2014). Evidence supports that MOR signaling in the NAc facilitates social reward, with intra-NAc morphine infusion increasing social play behavior that could be decreased by NLX (Trezza et al., 2011). MOR signaling in the NAc, is predominantly associated with reward processing (Shin et al., 2010; Nummenmaa et al., 2018). MOR agonists act on local inhibitory neurons in the VTA, causing disinhibition of (VTA^DA^) neurons and subsequently increased DA release into the NAc (Johnson and North, 1992; Pierce and Kumaresan, 2006). On the other hand, KORs in the NAc have been implicated in aversive, dysphoria-like behavior (Mucha et al., 1985; Pfeiffer et al., 1986; Shippenberg et al., 2007), and are thought to decrease DAergic NAc (NAc^DA^) signaling (Ehrich et al., 2014; Ebner et al., 2010). Chemogenetic inhibition of VTA^DA^ neurons preferentially targeting the NAc medial shell prevents the acquisition of heroin self-administration, supporting the idea that mesolimbic DA release is required for the reinforcing properties of opioids (Corre et al., 2018). MOR agonism may also regulate the reinforcement of rewards by promoting DA release through relieving GABA_B_-mediated inhibition of glutamatergic inputs to DA neurons (Chen et al., 2015).

Conversely, impaired maturation of the mesolimbic reward circuitry, marked by hypofunctioning VTA^DA^ neurons, has been linked to social deficits reversible by activation of glutamatergic signaling during the postnatal period or optogenetic-evoked DA release in the VTA in adulthood (Bariselli et al., 2016). Moreover, VTA hypoactivity has been associated with social deficits in female mice following adolescent JSI that could be reversed by chemogenetic activation (Tan et al., 2021; Supplementary Table S1). Provided that VTA^DA^ signaling is important for social reward processing, VTA circuitry changes driven by chronic opioid use may underlie low sociability following abstinence. A recent study identified MOR-expressing VTA neurons as facilitators of social deficits during protracted opioid withdrawal (Jo et al., 2024). While global MOR-KO mice did not show reduced sociability during fentanyl withdrawal, re-expression of MORs in the VTA of MOR-KO mice was sufficient to facilitate low sociability without affecting other withdrawal-related behaviors. This finding corroborates previous studies describing the role of MOR signaling in modulating social behavior (Moles et al., 2004; Becker et al., 2021) and characterizes the VTA as a complex neural substrate with distinct neuronal populations relevant to social processing. In this scenario, persistent adaptations in VTA neurons might work to alter the hedonic value of social interactions, with endogenous opioid release no longer promoting rewarding states induced by prosocial interactions. However, further studies are needed to elucidate the effects of chronic opioid use on VTA transmission on social behavior during protracted withdrawal.

Additional DA populations may play a role in social behavior. In contrast with VTA^DA^ activity, DAergic DRN (DRN^DA^) synapses appear to be potentiated following social isolation (Matthews et al., 2016). This DA population is hypothesized to recapitulate a “loneliness-like” state in which there is increased motivation to seek social contact concurrent with an aversive affective state. Activation of DRN^DA^ neurons is aversive in the absence of social stimuli, however, it increases social preference in the presence of a social target. Hence, whereas the VTA^DA^ population can facilitate prosocial behavior, DRN^DA^ neurons may promote the negative emotional state associated with SI. Expression of tryptophan hydroxylase (TPH2) defines another DRN population (Zhang et al., 2004). Repeated optogenetic activation of DRN^5-HT^ neurons, but not VTA^DA^ neurons, promotes long-lasting rescue of social preference in a mouse model of autism (Luo et al., 2017). Moreover, optogenetic inhibition of DRN^5-HT^ activity leads to low sociability, while activation of this population enhances prosocial behavior (Walsh et al., 2018). Given the converging evidence pointing to a role of 5-HT in prosocial behavior, it is unsurprising that decreased 5-HT release facilitates low sociability during protracted withdrawal (Valentinova et al., 2019; Pomrenze et al., 2022). Particularly, opioid modulation of DRN activity by the endogenous opioid dynorphin, which binds to Gi-coupled KORs, contributes to these social deficits through decreases in 5-HT release in the NAc (Pomrenze et al., 2022). Deletion of KORs from this cell population is enough to prevent low sociability during protracted withdrawal. Another study has shown that MOR KO in the DRN before heroin exposure prevents the emergence of social deficits during withdrawal (Lutz et al., 2014). Thus, it appears that chronic opioid use alters opioid transmission within major monoaminergic nuclei in the brain, potentially driving low sociability through MORs and KORs.

### 4.2 Cortex

The PFC plays high-level executive functions such as decision-making, problem-solving, emotional regulation, and social cognition. Impaired PFC function is believed to promote antisocial behaviors, including impulsive aggression and violence (Davidson et al., 2000). In adult mice, chronic isolation leads to PFC hypomyelination and decreased interaction time with conspecifics (Liu et al., 2012). Both behavioral and structural deficits could be reversed by social re-introduction. This brain region is typically divided into orbitofrontal (OFC), dorsolateral (dlPFC), ventrolateral (vlPFC), and medial (mPFC), which are collectively thought to modulate social processing (Ko, 2017). In humans, decreased PFC structure and function, including in the OFC, mPFC, and dlPFC subdivisions, has been correlated with antisocial behavior (Yang and Raine, 2009). Notably, early social deprivation appears to have long-lasting effects on PFC activity (Wang et al., 2022; Tan et al., 2021; Park et al., 2021). A recent study reports that JSI stress leads to hypofunctioning PFC pyramidal neuron activity that promotes aggression in males and social withdrawal in females (Tan et al., 2021). These prefrontal cortical circuits seem to be particularly affected by JSI with notable sex differences. In males, JSI has been shown to reduce the activity of PFC → basolateral amygdala (BLA) connections and result in disinhibition (increased BLA activity), promoting aggression. However, in females the activity of PFC → VTA connections is reduced, with reduced VTA^DA^ neuron activity, producing social withdrawal. JSI has also been shown to affect dendritic spine density PFC neurons, however, some groups report increased dendritic spine density while others report reduced density (McGrath and Briand, 2022; Wang et al., 2012). Others reported no significant changes in the number of dendritic spines but instead that the morphology of spines was affected and that there was an increased proportion of immature dendritic spines in JSI animals, coinciding with impaired LTP at BLA → mPFC synapses (Medendorp et al., 2018).

Abnormal PFC activity is a hallmark of addiction as well, with studies reporting baseline hypoactivity paired with hyperactivity in response to drug-related cues (Goldstein and Volkow, 2011). A recent study showed that chronic opioid self-administration gradually decreases mPFC basal activity, generating cognition deficits that are rescuable by chemogenetic activation (Anderson et al., 2021). Specifically, PFC projections to the NAc are thought to play an important role in control-reward circuits underlying opioid abuse. Inhibition of this projection has recently been shown to decrease cue-induced heroin reinstatement, modulating opioid-seeking during relapse (Clarke et al., 2024). A recent study has shown that opioid withdrawal reduces neuronal excitability of mPFC→NAc projections (Qu et al., 2020). mPFC→NAc projections also appear to be blunted after social deprivation, the activation of which seems to be particularly necessary for the social recognition of familiar conspecifics by JSI mice (Park et al., 2021). Given the converging evidence, impaired connectivity between the PFC and NAc may contribute to some of the social deficits associated with protracted opioid withdrawal. Morphine withdrawal has been shown to enhance dynorphin release in the mPFC and impair memory processing in mice (Abraham et al., 2021). KOR signaling in the mPFC reduces DA release and is required for KOR-mediated aversion (Tejeda et al., 2013), suggesting that enhanced KOR signaling during opioid abstinence may disrupt learning and other higher cognitive functions. mPFC→ paraventricular thalamus (PVT) projections have also been found to have reduced activity following SI immediately after weaning (Yamamuro et al., 2020). In adults, chemogenetic inhibition of this pathway is sufficient to generate low sociability, whereas activation of this pathway in JSI mice can rescue low sociability. Few studies have explored the role of opioid receptors in cortical structures in the context of OUD. A recent study reported that cognitive deficits following opioid abstinence are mediated by KOR activation in the PFC (Abraham et al., 2021). The ACC has also been implicated in the development of somatic symptoms of opioid withdrawal and hyperalgesia (McDevitt et al., 2021). Both human and rodent studies provide evidence for increased ACC activity during opioid withdrawal (Lowe et al., 2002; Chu et al., 2015), as well as increased glutamate release (Hermann et al., 2012; Hao et al., 2005).

The OFC has long been associated with social and affective regulation (Rempel-Clower, 2007; Rolls, 2004; Kuniishi et al., 2016; Downar, 2019). In humans, the OFC has been implicated in self-monitoring (Beer et al., 2006), social judgment (Willis et al., 2010), and social cognition (Cicerone, 1997). Patients with OFC damage show a reduced ability to identify their inappropriate social behavior (Beer et al., 2006), as well as impaired interpretation of negative emotional expressions (Willis et al., 2010) and social situations (Cicerone, 1997). In this manner, it seems that OFC activity is particularly important for processing knowledge of self and others. In preclinical and clinical settings, projections from the OFC to the BLA have been linked to social approach behavior and propensity to social anxiety (Li et al., 2021). Inhibition of mOFC→BLA projections generates low sociability, further characterizing the role of OFC in modulating social functions. Early social deprivation has been reported to reduce the AMPA/NMDA ratio in projections from the medial OFC to the BLA (Kuniishi et al., 2022). Optogenetic inhibition of this pathway led to sociability deficits in control mice that were comparable to those exhibited by JSI mice. In a recent study, NLX-treated individuals showed reduced OFC activity when presented with a social reward, suggesting that OFC opioid signaling is relevant for encoding the hedonic aspect of social stimuli (Massaccesi et al., 2024). In the context of OUD, OFC signaling is especially thought to be important for the incubation of craving, given that its activity progressively increases following opioid abstinence (Fanous et al., 2012; Sun et al., 2006). More studies are needed to determine the role of this substrate, if any, in social malfunctioning related to opioid withdrawal.

### 4.3 Habenula

The Hb has received increasing attention due to having one of the highest expressions of MORs in the brain. This brain region plays a crucial role in regulating aversive and reward-related behaviors, being implicated in the negative affective states associated with drug withdrawal and dependence, as well as depressive- and anxiety-like states (Boulos et al., 2020; Bailly et al., 2023; Xu et al., 2018; Cho et al., 2020). It is subdivided into two sub-structure**s**, the lateral and medial habenula (lHb and mHb, respectively). Targeting the habenular subregions is hindered by their small size, which has led studies to adopt a cell-type approach. For instance, expression of the B4 nicotinic receptor is restricted to the mHb (Shih et al., 2014). A recent study has shown that MOR expression in B4-mHb neurons is necessary for somatic withdrawal symptom manifestation during NLX-precipitated withdrawal (Boulos et al., 2020). MOR-expressing habenular neurons have also been shown to encode aversive states, as stimulation of this population leads to place avoidance (Bailly et al., 2023). Repeated opioid exposure increases the strength of inhibitory dMSN to habenula targeting globus pallidus neurons (GpH) projections, increases dMSN excitability and renders dMSN→GpH connections less sensitive to MOR-induced inhibition (Wang et al., 2023). In the same study, fentanyl self-administration also potentiated the strength of inhibitory dMSN to substantia nigra pars compacta (SNc) projections, reducing the baseline rate of DA neuron spontaneous firing, and enhancing the ability of dMSNs to suppress DAergic cell firing. Inhibition of these striatal MOR^+^ neurons during withdrawal reduced somatic and affective symptoms of withdrawal.

Deletion of MOR in habenular Chrnb4-positive neurons has been shown to generate significant social deficits regardless of previous exposure to opioids (Allain et al., 2022). Reduced LHb synaptic strength has been linked with social deficits in mice undergoing morphine withdrawal (Valentinova et al., 2019). Moreover, inhibition of LHb → DRN projections decreases sociability, further suggesting that habenular activity contributes to social behaviors. TNF receptor-1 (*Tnfr1*)-expressing LHb neurons appear to be necessary for the development of low sociability during morphine withdrawal (Valentinova et al., 2019). Cre-dependent Tnfr1 knockdown in this neural substrate prevents opioid abstinence-driven social deficits. These studies reveal an emerging and critical contribution for the habenula in mediating the rewarding and aversive affective components of the stages of opioid use and abstinence.

### 4.4 Amygdala

Amygdalar subregions, including the BLA, as well as the extended amygdala, i.e., central amygdala (CeA), bed nucleus of stria terminalis (BNST), and NAc integrate stress and reward pathways and are thought to contribute to the negative affective states and reinforcement that maintains opioid dependence (Janak and Tye, 2015; Wilson and McDonald, 2020). In clinical and preclinical studies, chronic opioid use has been reported to impair signaling between the amygdala and regions involved in motivation and reward processing, most notably the NAc (Upadhyay et al., 2010; Zan et al., 2021). The amygdala→NAc transmission regulates DA release, and decreased excitatory synaptic transmission in this pathway has been linked to the development of depressive-like states during morphine abstinence (Zan et al., 2021). Moreover, corticotropin-releasing hormone (CRH) plays a crucial role in opioid addiction by amplifying stress responses in the amygdala, contributing to negative emotional states, and reinforcing opioid-seeking behaviors during withdrawal (Iredale et al., 2000). A recent study has shown that the activity of CRH-expressing CeA neurons that project to the VTA is enhanced after chronic opioid use and mediates negative affect during withdrawal (Jiang et al., 2021). Morphine withdrawal has also been shown to enhance KOR-dynorphin signaling in the amygdala (Zan et al., 2021). While KOR^−/−^ mice do not show a depressive-like phenotype after opioid abstinence in the sucrose preference test (SPT) and tail-suspension test (TST), KOR re-expression in the amygdala of these mice is sufficient for the development of aversive states associated with withdrawal. The CeA is another MOR-dense substrate involved in opioid addictive behavior that sends projections to several reward-aversion centers, including the BNST (Koob, 2009; Beckerman and Glass, 2012). A recent study identified MOR-expressing CeA neurons as drivers of somatic withdrawal symptoms (Chaudun et al., 2024). Studies have also reported increased dynorphin mRNA in the BLA following chronic exposure to opioids (Zan et al., 2021; Solecki et al., 2009). BNST opioid signaling appears to facilitate drug-seeking behavior (Gyawali et al., 2020; Beckerman and Glass, 2012; Delfs et al., 2000), with intra-BNST administration of MOR antagonists suppressing opioid self-administration (Walker et al., 2000). Inhibitory enkephalinergic projections from the BNST to the VTA preferentially synapse onto GABAergic neurons in the VTA which express high levels of MOR (Kudo et al., 2014). These inputs are hypothesized to provide potent inhibition through fast GABA-mediated mechanisms and slower enkephalin-mediated inhibition. This would result in powerful and sustained disinhibition of VTA^DA^ neurons, which could explain how BNST→VTA circuits influence opioid reinforcement and promote opioid-seeking behavior.

The BLA is involved in emotional processing and stress responses, promoting affective deficits during protracted morphine withdrawal and driving retrieval of drug withdrawal memory (Deji et al., 2022; Guo et al., 2024). It also sends projections to the CeA another brain region with upregulated activity during opioid withdrawal (Chaudun et al., 2024; Hartley et al., 2019). Recent reports have linked BLA hyperactivity with social isolation-induced anxiety-like behaviors and increased levels of the metabotropic glutamate receptor 5 (mGluR5) (Lin et al., 2018). In this study, the administration of a mGluR5 antagonist rescued negative affect in the elevated-plus maze (EPM) and open-field (OF). Early social deprivation has also been shown to decrease amygdalar DA levels and alter dendritic morphology in BLA neurons (Wang et al., 2012). Open questions remain as to whether there are convergent or separate ensembles of neuronal circuits mediating both negative affect associated with withdrawal and reinforcement associated with acute opioid intoxication. Such questions could be approached by resolving cellular resolution using approaches such as miniscope recordings during the different phases of opioid intoxication and withdrawal.

## 5 Critical knowledge gaps

This review has explored the connections between ELA and OUD, investigating the overlapping cellular, molecular, and circuit-level changes that exist in the two models. The two seem to display disruptions in opioidergic and DA signaling in key reward-related structures, and several studies suggest that cortical regions and the mesolimbic DA system seem to be particularly vulnerable. Additional studies are needed to further characterize the relevance of specific MOR- and KOR-rich circuits in prosocial behavior, how endogenous and exogenous opioids affect the activity and function of these circuits under basal conditions, and which cells and circuits are particularly vulnerable to change following ELA. Future work should elucidate mechanisms underlying the altered opioid reward and function of the opioid system in ELA models, as well as how these differences affect addiction-related behaviors. Moreover, greater efforts are needed to elucidate transcriptional changes caused by both opioid use and childhood abuse in both animal models and humans, as existing data remains inconsistent (Falconnier et al., 2023). Several studies examined how ELA affects opioid reward, however another open question is how ELA affects withdrawal-related behaviors. It was noted that alterations in the *κ*-opioid system may underlie the strengthening of “anti-reward” systems during withdrawal and that ELA induces changes in the *κ*-opioid system, suggesting that withdrawal-related behaviors may be affected by ELA. Furthermore, efforts should be made to bridge the gap between ELA, opioid addiction studies, and social deficits during withdrawal.

This review identified cortical and monoamine circuits as being critical components of the pathology underlying both OUD and ELA. Future research is required to delineate how ELA and social isolation impacts reward circuit development, and particularly the development of DAergic circuits. DA signaling is critical to the reinforcement of drug seeking, and hypo-DAergic states are thought to underlie withdrawal behaviors, therefore the disruptions in DAergic signaling observed in ELA models are particularly relevant to our understanding of how ELA confers increased vulnerability to OUD. Furthermore, an investigation of how MOR signaling in the VTA mediates sociability deficits during withdrawal states and whether ELA exacerbates this phenomenon could prove to be quite beneficial to the field. The interaction between ELA and maturation of the 5-HT system and the subsequent effects on opioid reward-related behaviors also warrants further investigation. The 5-HT system is known to regulate sociability and was demonstrated to be modulated by the opioid system in the NAc, where dynorphin inhibited 5-HT release and led to sociability deficits in withdrawal (Pomrenze et al., 2022). Additionally, ELA is thought to impair the development of the 5-HT system (Malave et al., 2022). However, the synergistic effects of ELA and chronic opioid use on the function of the 5-HT system and how this confers risk for OUD and affects sociability during withdrawal has not been thoroughly explored but remains an important question. Finally, several MOR-rich regions including the amygdala and habenula have recently been shown to influence both affective behaviors and sociability during withdrawal states. It is essential for future works to clarify how these circuits are affected by both ELA and chronic opioid use, and whether opioid receptors are involved in mediating the aversive withdrawal states. Complementing self-administration studies with targeted manipulation strategies–such as chemo and optogenetic interventions–, as well as *in vivo* recordings, offers a powerful means to dissect the specific neural circuits and receptor systems facilitating opioid addiction. These techniques provide the precision required to dissect the complex interplay between ELA-induced changes and opioid reward pathways. There are significant methodological challenges that remain in studying the effects of ELA. Standardizing protocols such as MS and LBN is essential to ensure reproducibility and comparability across studies. Variability in these models can obscure results and hinder progress in understanding the nuanced effects of ELA. Addressing these methodological limitations will be critical for advancing the field and translating findings into effective prevention and treatment strategies for opioid addiction.

## 6 Conclusion

Rodent research has provided valuable insights into how early social deprivation influences brain areas, circuits, and networks relevant to reward processing. Impaired social bond formation and poor social networks during critical developmental periods are now established to impact reward circuitry maturation and vulnerability to addiction, including to OUD. Strong evidence suggests that this ELA subtype can modulate opioid-seeking behavior in both passive and self-administration paradigms. Such behavioral changes are accompanied by distinct and persistent changes in endogenous opioid release and opioid receptor expression. Moreover, significant progress has been made in identifying the neural substrates underlying negative affective states and social deficits following opioid abstinence. Long-term neuroadaptations in multiple brain reward (NAc, VTA, PFC, DRN) and aversion substrates (Hb, PVT, and amygdala) have now been identified both following early social deprivation and prolonged opioid use. Notably, changes in opioid transmission have been directly implicated in social deficits during protracted opioid abstinence. Continued exploration of the reciprocal relationship between social deprivation and opioid addiction will aid understanding of the long-term impact of social experiences on opioid abuse behaviors and ultimately inform more effective treatment strategies for OUD prevention and intervention strategies.
