# Supplementary material for: Opioidergic tuning of social attachment: reciprocal relationship between social deprivation and opioid abuse
Source: Front Neuroanat. 2025 Jan 23;18:1521016. doi: 10.3389/fnana.2024.1521016 (PMC11798945; doi:10.3389/fnana.2024.1521016)
Supplement: Supplementary file 1 [file Table_1.docx]

|  | **Deficit** | **Procedure** |
| --- | --- | --- |
| **Maternal care** | Dams spend more time caring for pups upon reunion and show increased nest-building behavior [(Millstein and Holmes, 2007)](https://sciwheel.com/work/citation?ids=1205717&pre=&suf=&sa=0) | Complete isolation 3h/day during light phase |
| **Anhedonia** | No change in SP test [(Harrison et al., 2014)](https://sciwheel.com/work/citation?ids=1205718&pre=&suf=&sa=0) | Unpredictable group-separation 3h/day on P1-P14 |
| **Anxiety-like** | Decreased OF center time [(Weiss et al., 2011)](https://sciwheel.com/work/citation?ids=579099&pre=&suf=&sa=0)  Decreased OF center time and open arm entries in the EPM [(Romeo et al., 2003)](https://sciwheel.com/work/citation?ids=3606327&pre=&suf=&sa=0)  No effect in EPM, LDB, or OF [(Millstein and Holmes, 2007)](https://sciwheel.com/work/citation?ids=1205717&pre=&suf=&sa=0)  Increased exploration in the EPM [(Venerosi et al., 2003)](https://sciwheel.com/work/citation?ids=16785050&pre=&suf=&sa=0)  No effect in EPM or OF [(Tan et al., 2017)](https://sciwheel.com/work/citation?ids=5762682&pre=&suf=&sa=0)  Decreased anxiety-like behavior in the EZM, but increased in the EPM [(Parfitt et al., 2007)](https://sciwheel.com/work/citation?ids=3606323&pre=&suf=&sa=0)  No change in OF and increased open arm time in the EPM [(van Heerden et al., 2010)](https://sciwheel.com/work/citation?ids=16785166&pre=&suf=&sa=0)  Increased OF center time [(Kember et al., 2012)](https://sciwheel.com/work/citation?ids=10018154&pre=&suf=&sa=0)  No change in OF and EZM [(Harrison et al., 2014)](https://sciwheel.com/work/citation?ids=1205718&pre=&suf=&sa=0) | Unpredictable group isolation 3h/day  Group isolation 3h/day at 1200h on P2-P14  Complete isolation 3h/day during the light phase on P0-P13  Group isolation 3h/day during light phase on P2-P14  Group isolation 3h/day at random points during the light phase  Group isolation 3h/day during the light phase on P1-P10  Group isolation 3h/day at noon on P9  Group isolation for 24h on P9  Unpredictable group-separation 3h/day on P1-P14 |
| **Distress-like** | No effect on percent immobility in the FST [(Millstein and Holmes, 2007)](https://sciwheel.com/work/citation?ids=1205717&pre=&suf=&sa=0)  No effect on immobility in TST and FST [(Tan et al., 2017; Savignac et al., 2011)](https://sciwheel.com/work/citation?ids=5762682,4952690&pre=&pre=&suf=&suf=&sa=0,0)  No change in FST [(Macrì and Laviola, 2004)](https://sciwheel.com/work/citation?ids=2719645&pre=&suf=&sa=0)  No change in FST [(Kember et al., 2012)](https://sciwheel.com/work/citation?ids=10018154&pre=&suf=&sa=0) | Complete isolation 3h/day during light phase on P0-P13  Group isolation 3h/day at random points during light phase  Single group isolation 24h MS on P12  Group isolation for 24h on P9 |
| **Fear learning** | No effect on fear learning [(Tan et al., 2017)](https://sciwheel.com/work/citation?ids=5762682&pre=&suf=&sa=0) | Group isolation 3h/day at random points during light phase |
| **Social behavior** | Decreased social investigation in the socio-sexual interaction test [(Macrì and Laviola, 2004)](https://sciwheel.com/work/citation?ids=2719645&pre=&suf=&sa=0)  Decreased social novelty preference [(Harrison et al., 2014)](https://sciwheel.com/work/citation?ids=1205718&pre=&suf=&sa=0) | Single group isolation 24h MS on P12  Unpredictable group-separation 3h/day on P1-P14 |
| **Pain** | Increased pain threshold in VF but not HP. Increased nociceptive WT behaviors in females [(Paniagua et al., 2020)](https://sciwheel.com/work/citation?ids=12854987&pre=&suf=&sa=0)  No change in VF and HP [(Paniagua et al., 2020)](https://sciwheel.com/work/citation?ids=12854987&pre=&suf=&sa=0)  No change in VF [(Mizoguchi et al., 2019)](https://sciwheel.com/work/citation?ids=5555673&pre=&suf=&sa=0) | Group isolation 4-8h/day on P2-P17  Group isolation 3h/day on P2-P17  Group isolation 3h/day n P1-P21 |

**Supplemental Table 1. Summary table of behavioral changes observed following distinct MS protocols in mice.** Abbreviations: SP: sucrose preference, OF: open-field, EPM: elevated-plus maze, LDB: light-dark box, EZM: elevated zero maze, NIH: novelty-induced hypophagia, TST: tail suspension test, FST: forced-swim test, VF: Von Frey, HP: hot plate.
